# The ignitability, fuel ratio and ash fusion temperatures of torrefied woody biomass

**DOI:** 10.1016/j.heliyon.2020.e03582

**Published:** 2020-03-12

**Authors:** A.A. Adeleke, J.K. Odusote, P.P. Ikubanni, O.A. Lasode, M. Malathi, D. Paswan

**Affiliations:** a Department of Mechanical Engineering, Landmark University, Omu Aran, Nigeria; b Department of Materials and Metallurgical Engineering, University of Ilorin, Nigeria; c Department of Mechanical Engineering, University of Ilorin, Nigeria; d Metal Extraction and Recycling Division, National Metallurgical Laboratory, Jamshedpur, India

**Keywords:** Energy, Materials science, Teak wood, Melina wood, Torrefaction, Ignitability index, Fuel ratio, Ash fusion temperatures

## Abstract

The impact of torrefaction temperature on the ignitability, fuel ratio and ash fusion temperatures of two tropical deciduous woods (Teak and Melina) were investigated in a setup of tubular furnace. The properties considered are calorific value, fuel ratio, ignitability index, ash compositions and ash fusion temperatures of the biomass. Six different temperatures (220, 240, 260, 280, 300 and 320 °C) at 60 min reaction time were considered. The results indicated that as torrefaction temperature increased, the calorific value, fuel ratio and ignitability index of the biomass also increased. The ignitability index of biomass (40–63) was better than the value (35) recommended for fuel applicable in thermal plants for power generation. The ash compositional analysis revealed that there was no variation in the quantity of SiO_2_, Al_2_O_3_, CaO along with other minerals for the raw and torrefied biomass. This implied that the temperature up to 320 °C has no significant impact on the compositions of biomass ash during torrefaction. The ash fusion temperature test showed that the biomass ash softens at ≈ 1200 °C and finally fused at ≈ 1300 °C. The study concluded that an increase in torrefaction temperature increases the thermal properties of the torrefied biomass without affecting the compositions of biomass ash or lowering the ash fusion temperatures.

## 1 Introduction

Fossil fuels such as coal, oil, and natural gas contribute 80% of more than 400 EJ of energy consumed in a year worldwide [1, 2, 3, 4]. These sources of energy are expected to deplete within the next 40–50 years [3]. In another report, the current reserves of oil, coal, and natural gas have been evaluated that it will last for the next 40, 60 and 150 years, respectively [5]. Irrespective of when the reserve will be exhausted, the present effect their usage plays in anthropogenic emission on the global climate is overwhelming [6]. The increasing rise in global temperature due to the use of fossil fuels continues to be scrutinized. The intergovernmental panel on climate change (IPCC) reported that continued emissions from fossil fuels will lead to a temperature increase of 1.4 and 5.8 for 1990 to 2100 [3,7]. Therefore, to avoid catastrophic consequences on the globe, research on the use of biomass (a carbon-neutral material) as a renewable fuel to be co-fired with coal in thermal plants for power generation continues to gain popularity worldwide [2, 8, 9, 10, 11, 12]. Nigeria as a country have not taken full advantage of woody biomass though a large proportion (91.1 million tons) of it are constantly wasted in the Ilorin metropolis daily [13]. The experience of wastage of biomass materials is similar all over the nation where higher volume are been generated at various wood processing industries. Two of the commonly used deciduous woody biomass in the country are Teak and Melina woods [14]. Their use contributes largely to waste pile which are often burnt or disposed into flowing streams. These woody biomass wastes can successfully be utilized with coal in thermal plants though limited to various technical issues. The direct comparison of biomass with coal as solid fuel in electricity and heat generation often reveals inferior properties of biomass such as low energy content, high moisture and poor ash fusion temperatures among others [15]. However, these limitations and challenges can be overcome by thermally treating biomass using the torrefaction process. Torrefaction process involves thermal treatment of biomass in an inert/oxygen-deficient environment at a temperature of 200–350 °C [16, 17]. This process has previously been used to improve the fuel properties of *Terminalia ivorensis* [8], reed canary grass, wheat straw and willow wood [15], oil palm [9], Poplar and Spruce woods [18], oil palm fiber and eucalyptus [19] and sewage sludge [20]. However, most of these works do not pay significant attention to the fuel ratio and ignitability with the heating value (calorific value) of biomass chars produced through torrefaction. Research work on the changes in the ash compositions and ash fusion temperatures of the torrefied biomass of Nigeria origin is also limited. The majority of research to-date on torrefaction of woody biomass is traceable to other nations and the few studies in Nigeria have not addressed comprehensively, the impact of torrefaction temperature on the calorific value, fuel ratio and ignitability of tropical deciduous woody biomass of Nigeria origin. Therefore, the present work presents a fundamental investigation on the impact of torrefaction temperature on thermal properties and ash analyses of tropical deciduous wood that are key characteristics of fuel used for power generation in thermal plants.

## 2 Methodology

### 2.1 Materials

Teak and Melina wood lumbers were obtained from Benin, Nigeria (6° 20ˊ 17.34ʺ N, 5° 37ˊ 32.70ʺ E). They were pulverized using a Thomas Wiley Laboratory Mill (Model 4) and screened to obtain particles with a size <6.35 mm. The pulverized samples were sun-dried for five days (6 h/day) to remove surface and residual water and then stored in zip-locked polyethylene bags at room temperature for analyses and torrefaction experiment.

### 2.2 Thermogravimetric analyses and torrefaction experiment

**Figure 1:**
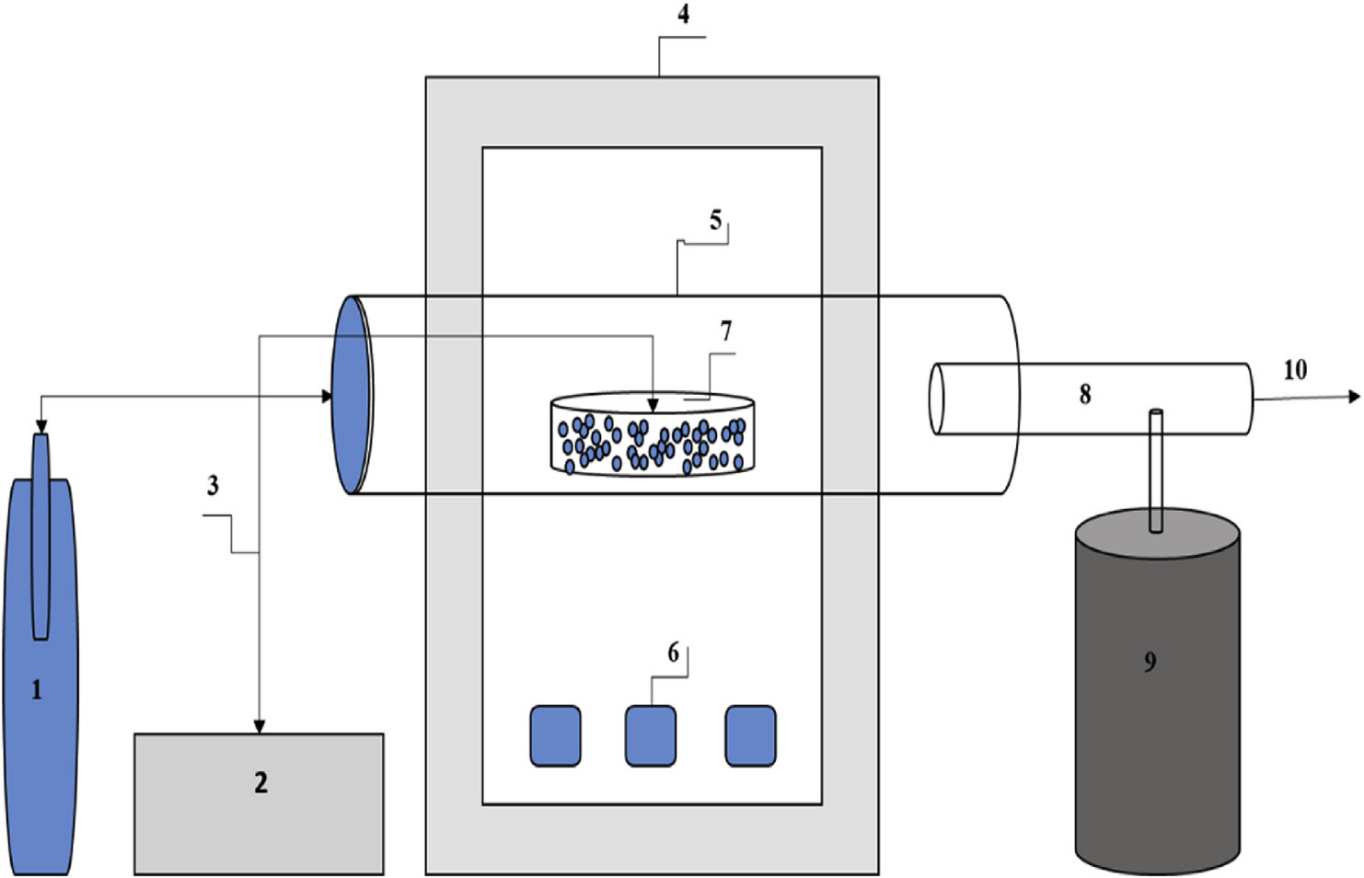
Schematic diagram of the torrefaction experiment. 1- Nitrogen cylinder; 2, external indicator; 3, external thermocouple (K-type); 4, tubular furnace; 5, heating chamber; 6, temperature controller and display unit; 7, Pulverized biomass in metallic crucible; 8, outlet pipe; 9, water bath for condensable exhaust; and 10, non-condensable gases.

Thermogravimetric measurement was carried on the Teak and Melina wood fine samples using a thermogravimetric analyzer (Perkin Elmer TGA-7, Massachusetts, USA, 0.1K/min precision) with N2 purge gas (100 mL/min). Samples (5 mg) were analyzed at linear heating rates of 5/min. The experiments were carried out under non-isothermal conditions from 30 to 400. The laboratory-scale torrefaction unit used in this study is shown in the schematic diagram (Figure 1). During torrefaction, 46 g of wood fines were heated under a continuous N2 flow to the desired temperature (220, 240, 260, 280, 300 or 320 °C) at a heating rate of 12 °C/min. The samples were held at that temperature for a residence time of 60 min as recommended by [21, 22, 23].

### 2.3 Proximate, ultimate and calorific value analyses

The moisture content (MC) was determined according to the ASTM E871-82 standard [24] in an oven (Model No: OF-22G, JESO TECH, Korea). Volatile matter (VM) contents of raw Teak and Melina woods were determined according to BS EN 15148 standard [25]. The ash content (AC) of raw Teak and Melina woods were carried out in a muffle furnace (Model No: CBFL518C, USA) following the ASTM E1755-01 standard [26]. The proximate analyses of torrefied Teak and Melina were carried out according to IS: 1350-1 standards [27] in an oven and a muffle furnace. The analyses were carried out in duplicates and the average has been reported. Fixed carbon (FC) content was obtained by difference, for both raw and torrefied Teak and Melina. The CHN analysis of the raw and torrefied biomass was carried out in a LECO-CHN628 Analyzer (Model No: 622-000-000, SN-12357) using ASTM D5373 standard [28]. Sulfur analysis was carried out in a LECO S-144DR Sulphur Determinator (Model No: 606-000-300, SN-477, 0.3% accuracy) using ASTM D4239-11 standard [29]. Oxygen contents for the raw and torrefied biomass were calculated by difference, which is 100 - (C% + H% + N% + S%+ Ash %). The calorific values for both raw and torrefied samples were determined in a Parr 6200 Oxygen Bomb Calorimeter (Model No: A1290DDEE; 001 °C reading accuracy) following ASTM D5865-04 standard [30].

### 2.4 Evaluation of fuel ratio and ignitability index

The fuel ratio $\left({\text{F}}_{\text{R}}\right)$ of the raw and torrefied biomass was evaluated using Eq. (1) while the ignitability index (${\text{I}}_{\text{I}}$) was calculated using Eq. (2) [31].

(1) $${\text{F}}_{\text{R}}=\frac{\text{FC}}{\text{VM}}$$

(2) II=(CV−81FC)(VM+MC)

where CV is the calorific value in kJ/kg, FC is the fixed carbon (%), VM is the volatile matter (%) and MC is the moisture content.

### 2.5 Ash composition and ash fusion temperature analyses

Muffle furnace at 815 °C for 3h was used to ash the pulverized Teak (Tw) and Melina (Mw) wood samples. The ash compositional analysis of Teak and Melina was carried out using an X-ray fluorescence (XRF) spectrometer (Bruker S8 TIGER model, 0.3% accuracy). Ash of 8 g was thoroughly mixed with 2 g of wax (binder). The mixture was made into a pellet of 34 mm diameter (1 mm thick). The sample was then placed in the sample holder and transfer into the XRF for analyses. Spectra plus launcher was used to collect ash compositions. Ash fusion temperature (AFT) analyses were carried out following the ASTM-D 1857-04 standard [32]. 1 g of the ash was mixed with dextrin solution to be formable into a cone in shape plate. The formed cones were allowed to dry for 4–5 min in the sample holder. The AFT (AF700) furnace was purged with N_2_ and O_2_ gases (50/50) at 2.2–2.5 L/min. At 400 °C, the sample was placed in the furnace while the process was monitored with AFT700 software to evaluate the initial deformation temperature (DT), softening temperature (ST), hemispherical temperature (HT) and final fusion temperature (FT).

## 3 Results and discussion

### 3.1 Thermogravimetric assessment of teak and melina woods

**Figure 2:**
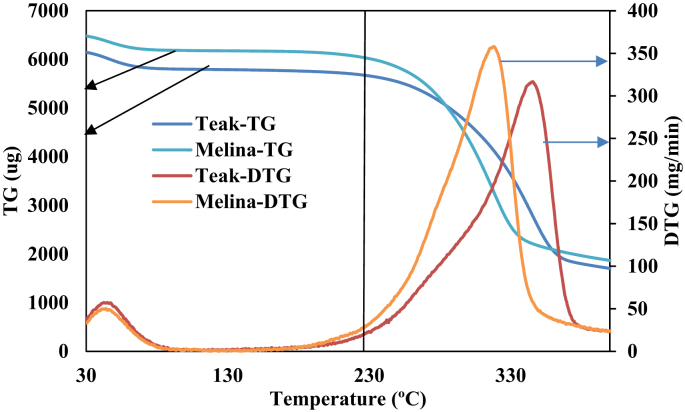
Thermogravimetric (TG) and first derivative thermogravimetric (DTG) curves for teak and melina woods at 5 °C/min heating rate ramped from 30 at 400 °C.

Figure 2 shows the weight loss curves (TG and DTG) of the Teak and Melina wood samples obtained from a non-isothermal TGA experiment. Aside from the initial weight loss between 30-100 which represents the evolution of preliminary unbounded moisture [8, 23], weight loss in the biomass was relatively small up to 220. This temperature has been reported to be the point at which thermal decomposition begins to occur in woody biomass [33] and this was depicted by the TG and DTG shown in Figure 2. A significant drop in weight was observed from 220 to 380 °C arising from hemicellulose, cellulose and lignin decompositions [34]. However, above 380, the weight loss was not as significant as the previous stage. Thus, the torrefaction process used in this study for Teak and Melina woods was bounded between 220 and 320 °C. The temperature (320 °C) was the point where the highest weight loss was recorded for both teak and Melina wood. The present study aims to produce biochar with a high fuel ratio and improved ignitability while reducing the volatile matter contents in the biomass. It is therefore important to carefully select temperature which has been reported to have the largest influence on the properties of biomass when subjected to the torrefaction process [21, 22, 23]. Thus, to obtain the desired fuel ratio, ignitability, and energy contents, torrefaction must be carried out between 220 and 320 °C.

### 3.2 Combustion characteristics of raw biomass and chars

**Figure 3:**
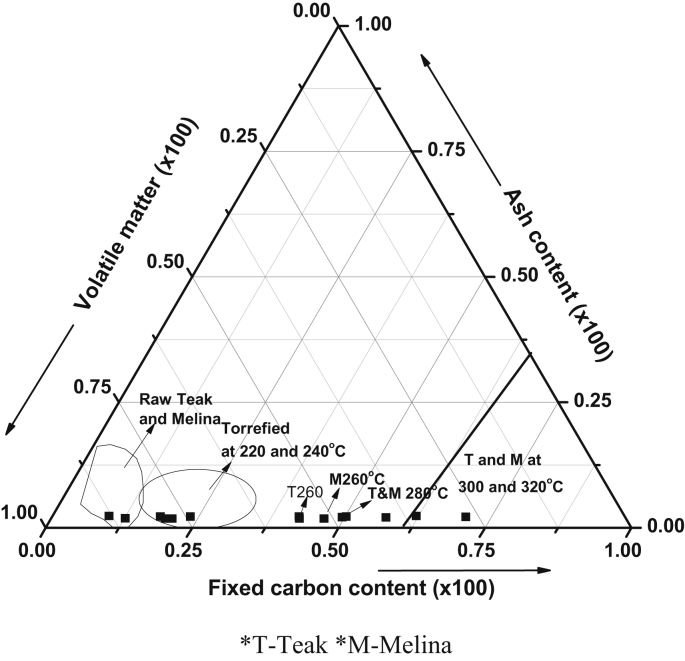
Classification of raw and torrefied biomass based on the proximate characteristics.

**Figure 4:**
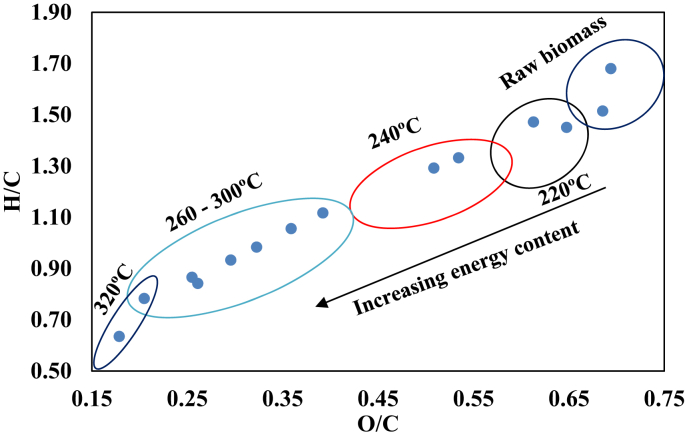
H/C and O/C atomic ratios for raw and torrefied woody biomass.

The combustion characteristics (proximate, ultimate and calorific values) are presented in Table 1. The moisture content of the raw Teak and Melina woods was 7.23 and 7.53%, respectively. It was observed that the torrefaction process led to a reduction to 4% at 220 °C which continued to reduce as the temperature increases. The volatile matter also reduced from 79 - 81% for raw biomass to 40 - 26% for the samples torrefied at 320 °C. High volatile matter (VM) contents in biomass often lead to rapid and difficult combustion. A bigger reactor volume is also usually required to prevent high rate of pollutant emissions, unburnt products, and polyaromatic hydrocarbons during the combustion of high VM biomass [17, 35]. Thus, the reduction in the VM content is a welcome development for these solid fuels that are usable in an existing coal-fired plant. This is a result of the reduced VM which could encourage an accurate synergy during co-firing of the torrefied biomass (300 and 320 °C) with coal. A more stable flame and complete combustion is possible with the Teak and Melina wood dusts torrefied at 300 and 320 °C [6]. Figure 3 shows that at increased temperatures (300 and 320 °C), the FC was high while ash content remains relatively constant at all temperatures (Table 1). By implication, at higher temperatures, a higher FC is obtainable [35]. However, the FC contents in Melina wood were higher than that of teak wood at the same temperatures. The calorific value also shows that Melina wood was better improved than teak wood at each torrefaction temperature. The highest calorific value (31.08 MJ/kg) was obtained from the sample with the least VM content (26.86%). The highly reactive biomass was denatured to give a more stable fuel. Thus, operating at 300 and 320 °C are the best in terms of the proximate content of the fuel. This trend was a repetitive one with the ultimate analyses. Table 1 shows that the carbon content increased from 47.84 to 68.70% (320 °C) for Teak wood. Similarly, it increased from 47.09 to 75.34% (320 °C) for the Melina wood. It could be observed that C increased with torrefaction temperature. The lower carbon and higher O and H contents in the biomass often leads to a reduced energy value since there is a lower energy stored in the CO and CH bonds than CC bonds [17]. A typical decrease in H/C and O/C ratio shown in Figure 4 implied that there is an increase in the aromaticity and a reduction in oxygen-containing hydroxyl, carboxyl, ether, and ketone functional groups in the biomass. Thus, an increase in energy density as torrefaction temperature increases [6]. Therefore, the torrefaction of biomass at 300 and 320 °C gave the best output in terms of the combustion characteristic suitable for co-firing with coal in existing co-fired plants.

**Table 1:** Proximate, ultimate and calorific value characteristics of the raw and torrefied biomass.

Sample	MC (%)	VM (%)	AC (%)	FC (%)	C (%)	H (%)	N (%)	S (%)	O (%)	CV (MJ/kg)
T_w_	7.23	79.26	1.73	11.73	47.84	6.09	0.39	0.26	43.69	18.71
220	4.00	75.53	1.76	18.71	49.20	6.00	0.37	0.22	42.45	19.97
240	3.22	74.98	1.79	20.02	54.78	5.95	0.36	0.22	37.11	21.68
260	2.74	54.20	1.78	41.28	60.40	5.67	0.35	0.21	31.56	23.61
280	2.60	50.22	1.77	45.41	62.33	5.53	0.35	0.21	29.81	24.64
300	2.56	47.08	2.05	48.32	63.99	5.29	0.35	0.22	27.51	26.44
320	2.01	40.04	2.00	55.95	68.70	4.86	0.34	0.21	23.89	28.86
M_w_	7.52	81.42	2.15	8.92	47.09	6.65	0.38	0.24	43.54	18.37
220	3.88	76.22	2.17	17.73	50.11	6.20	0.38	0.22	40.92	20.03
240	3.01	71.89	2.17	22.89	53.86	6.03	0.20	0.20	38.35	21.07
260	2.68	54.09	2.17	41.06	66.05	5.18	0.36	0.22	26.02	23.44
280	2.60	46.33	2.18	48.89	68.86	5.01	0.34	0.22	23.39	25.25
300	2.53	34.64	2.29	60.55	72.04	4.74	0.33	0.20	19.67	29.09
320	1.15	26.86	2.17	69.82	75.34	4.02	0.32	0.21	17.94	31.08

### 3.3 Fuel ratio and ignitability index

**Figure 5:**
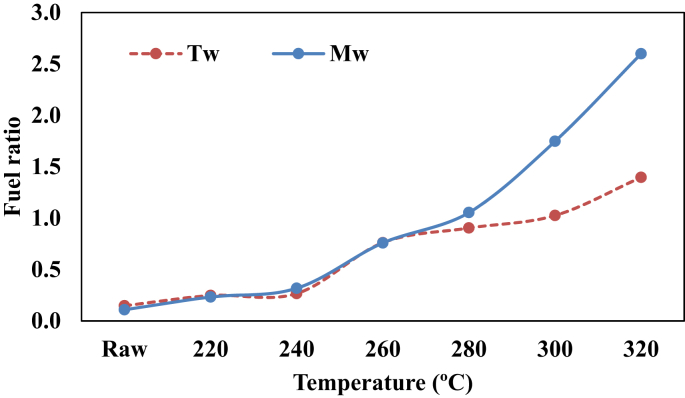
Influence of temperature on the fuel ratio of Teak and Melina woods.

**Figure 6:**
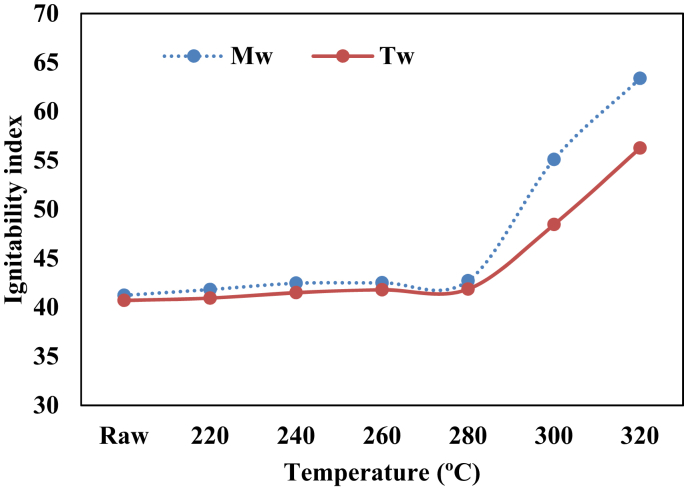
The ignitability index of raw and torrefied biomass based on temperature variation.

**Figure 7:**
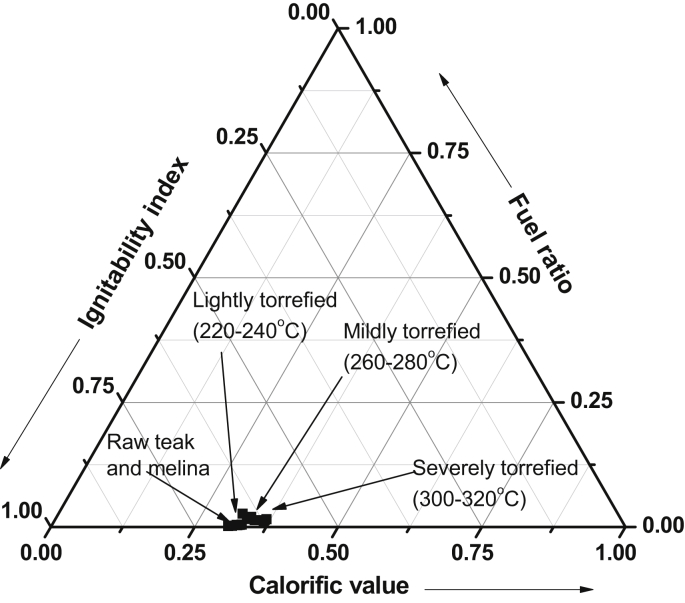
Positions of raw and torrefied biomass in the combustion classification based on calorific value, ignitability index and fuel ratio.

Other useful parameters to determine the quality of a solid fuel is the ratio of its fixed carbon to volatile matter [36]. The fuel ratios of the raw and torrefied biomass samples are presented in Figure 5. It can be observed that the fuel ratios of the torrefied samples were higher than the raw sample. This resulted from the loss of volatile matters of the raw biomass during the torrefaction process. A low fuel ratio usually results in more flaming combustions, less char combustion, and quicker burnout. However, the torrefaction process increased the fuel ratio which will enable a stable and lasting combustion process, especially when used in a boiler [6, 31]. Melina gave the highest fuel ratio at 300 °C (1.75) and 320 °C (2.60) compared to Teak wood which the fuel ratio at 300 °C was 1.03 and at 320 °C was 1.40. The ignitability indices of Melina and Teak woods are presented in Figure 6. The ignitability index is also an indication of the likely performance of torrefied biomass in the furnace/boiler conditions [37]. The ignitability indices of the torrefied biomass were better than those of raw samples and it increases with torrefaction temperature. It has been previously reported that a high fuel ratio significantly points to a high ignitability index. This was confirmed in the present study (Figure 7). Asthana [31] opined that when the ignitability index of solid fuel is less than 35, it might be difficult to utilize such fuel efficiently in a boiler. However, the sample torrefied at 300 and 320 °C surpassed this benchmark distinctly for both Teak (48 and 56) and Melina (55 and 63) woods, respectively.

### 3.4 Ash composition and ash fusion temperatures analyses

The ash compositions of the raw and torrefied biomass are presented in Table 2. The ash contained SiO_2_, Al_2_O_3_, TiO_2_, CaO, and K_2_O among others. It was observed that the ash composition was not significantly affected by the torrefaction temperature. The SiO_2_, CaO and K_2_O contents of the biomass were higher than the other chemical constituents. The percentage of SiO_2_ present in Melina and Teak woods is lower than what was reported by Demirbas [38] for Red oak wood (49.0%), wheat straw (48.0%) and Hazel nutshell (33.7%). However, the Al_2_O_3_ contents were in the same range whereas the percentage CaO range obtained in the present study was higher than the range of 3.7–17.5% reported for the biomass. The other oxides were reported with different degrees of variations including the K_2_O content. The silicate (SiO_2_) content of the biomass shows that there will be less erosion-abrasion corrosion and slagging problems during its utilization as a solid fuel for thermal application [6]. It has been reported that higher content above 35% of silica minerals such as quartz, tridymite, and cristobalite could increase the wear of the combustion chamber [39]. Hence, the usage of the biomass (Teak and Melina) in this present study will have a lower tendency of wearing the combustion chamber since the silica mineral present in the samples are lower than 35%. The amount of silica content present in the biomass will also limit the formation of low-temperature alkaline silicates that can rapidly increase slagging and even health risks. The CaO and MgO contents of fuel ash have also been reported to affect slagging, fouling and corrosion. Biomass content with high contents of CaO and MgO exhibits manageable slagging, fouling and corrosion problems. The tendency of Melina and Teak woods to reduce lime/calcite usage and plant operation cost of an installed fuel gas desulphurization system during acid gas abatement is high. This is because of the high CaO and MgO content in its ash. Compared to coal, the raw and torrefied biomass contained lower SO_3_ and Al_2_O_3_. However, the lower content of TiO_2_, Fe_2_O_3_, and Na_2_O could account for lower ash fusion temperature (AFTs) compared to coal [40]. The AFTs of raw and torrefied biomass samples are presented in Table 3. The AFTs show that the initial deformation temperature (DT), softening temperature (ST), hemispherical temperature (HT), and final fusion temperature (FT) of Teak were higher than those of Melina. Though, the Teak and Melina woods used in this study have desirable AFT which may be because of Ca, Al and Ti-bearing minerals present in them. The lower content of these minerals could be responsible for the rapid decrease in the AFTs of some biomass such as rice husk, wheat husk and red oak wood [38]. The range of FT for the ash (1290–1300 °C) obtained for the samples in the present study is comparable to the ash of some coal used in fluidized-bed combustor [41]. The temperature range utilized for the mild pyrolytic treatment of the Teak and Melina woods does not affect the ash composition and ash fusion temperature negatively. Thus, based on the ash composition, AFTs, and other characterization at 300 and 320 °C, the torrefied Teak and Melina are suitable for energy generation in thermal plants.

**Table 2:** Ash analyses for the raw and torrefied Teak and Melina woods.

Samples/Temp. (°C)	Oxides (%)										Sum
	SiO_2_	Al_2_O_3_	TiO_2_	Fe_2_O_3_	CaO	MgO	Na_2_O	K_2_O	SO_3_	P_2_O_5_	
T_w_	30.34	4.86	0.30	3.60	26.82	5.42	3.00	18.28	3.20	4.18	100
220	30.24	4.84	0.50	3.58	26.80	5.40	3.01	18.26	3.22	4.15	100
240	30.19	5.00	0.41	3.42	26.82	5.45	3.02	18.18	3.23	4.28	100
260	30.22	4.84	0.40	3.62	26.8	5.43	2.99	18.30	3.20	4.20	100
280	30.26	4.82	0.39	3.61	26.70	5.42	2.97	18.46	3.21	4.16	100
300	30.25	4.83	0.39	3.64	26.71	5.42	2.97	18.40	3.21	4.18	100
320	30.34	4.86	0.30	3.60	26.82	5.42	3.00	18.28	3.20	4.18	100
M_w_	31.68	4.20	0.28	3.28	27.02	5.26	2.86	18.11	3.24	4.07	100
220	31.50	4.20	0.48	3.48	26.98	5.22	2.83	18.09	3.22	4.00	100
240	31.68	4.20	0.28	3.28	27.02	5.26	2.86	18.11	3.24	4.07	100
260	31.58	4.23	0.39	3.26	27.01	5.23	2.87	18.12	3.26	4.05	100
280	31.56	4.22	0.40	3.22	27.03	5.21	2.90	18.11	3.27	4.08	100
300	31.49	4.20	0.42	3.21	27.00	5.24	2.91	18.15	3.28	4.10	100
320	31.68	4.20	0.28	3.28	27.02	5.26	2.86	18.11	3.24	4.07	100

**Table 3:** Ash fusion temperature of raw and torrefied biomass.

Samples/Temp. (°C)	DT (°C)	ST (°C)	HT (°C)	FT (°C)
T_w_	1170	1200	1240	1320
220	1200	1210	1230	1320
240	1200	1220	1230	1320
260	1200	1220	1230	1320
280	1200	1220	1230	1320
300	1200	1220	1230	1320
320	1210	1220	1230	1310
M_w_	1100	1180	1220	1300
220	1100	1200	1220	1290
240	1120	1200	1210	1300
260	1120	1200	1210	1300
280	1120	1200	1210	1300
300	1120	1200	1210	1300
320	1120	1200	1220	1300

∗DT-Initial deformation temperature; ST-softening temperature; HT-hemispherical temperature; FT-fusion temperature.

## 4 Conclusion

The impact of temperature on the ignitability, fuel ratio and ash fusion temperature of Teak and Melina woods were investigated. An increase in torrefaction temperature led to the increase in the ignitability index, fuel ratio and the energy content of the biomass. Melina wood was better improved compared to Teak in terms of fuel ratio, ignitability index, and energy content. The variation in the SiO_2_, Al_2_O_3_, CaO, MgO and other minerals was negligible in the raw and torrefied Teak and Melina. The ash of the biomass softened at 1200 °C and finally fused at 1300 °C. Therefore, the increase in temperature during torrefaction increased the thermal properties of the biomass solid fuel without affecting the ash composition or lowering the ash fusion temperature. These solid fuels properties met with the conditions required for energy generation in thermal plants.

## Declarations

### Author contribution statement

Adeleke, A.A: Conceived and designed the experiments; Performed the experiments; Wrote the paper.

Odusote, J.K.: Conceived and designed the experiments.

Ikubanni, P.P.: Contributed reagents, materials, analysis tools or data.

Lasode O.A.: Analyzed and interpreted the data.

Malathi, M. & Paswan, D.: Performed the experiments.

### Funding statement

This work was supported by The World Academy of Science (TWAS Award No: FR: 3240287331) & The Council of Scientific and Industrial Research (CSIR FUND: P-81-1-09).

### Competing interest statement

The authors declare no conflict of interest.

### Additional information

No additional information is available for this paper.
